# A genetic variant in *IL-6* lowering its expression is protective for critical patients with COVID-19

**DOI:** 10.1038/s41392-022-00923-1

**Published:** 2022-04-02

**Authors:** Bo Gong, Lulin Huang, Yongquan He, Wen Xie, Yi Yin, Yi Shi, Jialing Xiao, Ling Zhong, Yi Zhang, Zhilin Jiang, Fang Hao, Yu Zhou, Huan Li, Li Jiang, Xingxiang Yang, Xiangrong Song, Yan Kang, Lin Tuo, Yi Huang, Ping Shuai, Yuping Liu, Fang Zheng, Zhenglin Yang

**Affiliations:** 1 Department of Health Management, Sichuan Academy of Medical Sciences & Sichuan Provincial People’s Hospital, University of Electronic Science and Technology of China, Chengdu, Sichuan China; 2 Sichuan Provincial Key Laboratory for Human Disease Gene Study, Center for Medical Genetics, Department of laboratory medicine, Sichuan Academy of Medical Sciences & Sichuan Provincial People’s Hospital, University of Electronic Science and Technology of China, Chengdu, Sichuan China; 3 Research Unit for Blindness Prevention of Chinese Academy of Medical Sciences (2019RU026), Sichuan Academy of Medical Sciences & Sichuan Provincial People’s Hospital, University of Electronic Science and Technology of China, Chengdu, Sichuan China; 4 Institute of Chengdu Biology, Sichuan Translational Medicine Hospital, Chinese Academy of Sciences, Chengdu, Sichuan China; 5 Center for Gene Diagnosis & Department of Laboratory Medicine, Zhongnan Hospital of Wuhan University, Wuhan, China; 6 Department of Laboratory Medicine, Wuhan Leishenshan Hospital, Wuhan, Hubei China; 7 Infectious Disease Department, Sichuan Provincial People’s Hospital, University of Electronic Science and Technology of China, Chengdu, 610041 China; 8 Department of Critical Care Medicine, Frontiers Science Center for Disease-related Molecular Network, State Key Laboratory of Biotherapy, West China Hospital, Sichuan University, Chengdu, China

**Keywords:** Infectious diseases, Medical genetics

## Abstract

Critical coronavirus disease 2019 (COVID-19) is associated with high mortality and potential genetic factors have been reported to be involved in the development of critical COVID-19. We performed a genome-wide association study to identify the genetic factors responsible for developing critical COVID-19. 632 critical patients with COVID-19 and 3021 healthy controls from the Chinese population were recruited. First, we identified a genome-wide significant difference of *IL-6* rs2069837 (*p* = 9.73 × 10^−15^, OR = 0.41) between 437 critical patients with COVID-19 and 2551 normal controls in the discovery cohort. When replicated these findings in a set of 195 patients with critical COVID-19 and 470 healthy controls, we detected significant association of rs2069837 with COVID-19 (*p* = 8.89 × 10^−3^, OR = 0.67). This variant surpassed the formal threshold for genome-wide significance (combined *p* = 4.64 × 10^−16^, OR = 0.49). Further analysis revealed that there was a significantly stronger expression of IL-6 in the serum from patients with critical COVID-19 than in that from patients with asymptomatic COVID-19. An in vitro assay showed that the A to G allele changes in rs2069837 within IL-6 obviously decreased the luciferase expression activity. When analyzing the effect of this variant on the IL-6 in the serum based on the rs2069837 genotype, we found that the A to G variation in rs2069837 decreased the expression of IL-6, especially in the male. Overall, we identified a genetic variant in *IL-6* that protects against critical conditions with COVID-19 though decreasing IL-6 expression in the serum.

## Introduction

In late December 2019, the novel coronavirus (SARS-CoV-2) infection causing coronavirus disease 2019 (COVID-19) was first reported in Wuhan, China, and quickly spread worldwide. As of May 28, 2021, SARS-CoV-2 had infected more than 100 million patients in 100 other countries, with total deaths exceeding 3 million (https://covid19.who.int/). The clinical features of COVID-19 are highly heterogenous, from death to mild symptoms, or even to no symptoms. The symptoms of COVID-19 include fever, myalgia, fatigue, dry cough, shortness of breath, sputum production, headache, hemoptysis, sore throat, and diarrhea. Lymphopenia, prolonged prothrombin time, and elevated lactate dehydrogenase levels have also been observed in patients who have COVID-19^1^. A computed tomography (CT) scan can identify bilateral patchy shadows or ground-glass opacity in the lungs of patients with COVID-19^1,2^. The mortality rates are driven predominantly by the subgroup of patients who have severe respiratory failure related to interstitial pneumonia in both lungs and acute respiratory distress syndrome. Severe COVID-19 with respiratory failure requires early and prolonged support by mechanical ventilation. However, the pathogenesis of critical COVID-19 and the associated respiratory failure remains unclear and there is no specific cure for the disease^3^.

Cohort studies have identified older age, male sex, and comorbidities such as hypertension, diabetes, and obesity as risk factors that predispose people to severe COVID-19^4,5^. In an analysis using rapid whole-exome sequencing of the data from four young male patients with severe COVID-19, rare putative loss-of-function variants of X-chromosomal *TLR7* were identified as the most biologically plausible candidates for causing critical COVID-19^6^. The study showed that a genetic predisposition might be associated with primary immune deficiencies among young patients with COVID-19. A genome-wide association study (GWAS) involving 1,980 patients with COVID-19 and severe disease (defined as respiratory failure) at seven hospitals in the Italian and Spanish epicenters of the SARS-CoV-2 pandemic in Europe was performed to delineate the host genetic factors that contribute to severe COVID-19 accompanied by respiratory failure^4^. The study detected cross-replicating associations with rs11385942 at locus 3p21.31 and with rs657152 at locus 9q34.2 and confirmed the potential involvement of the ABO blood group system, but the frequency of rs11385942 was almost zero in East Asians. Another GWAS on 2244 critically ill COVID-19 patients from 208 intensive care units (ICUs) in the United Kongdom (UK) identified and replicated novel genome-wide significant associations at four loci^7^. Numerous reports over the past year have described monogenic inborn errors of immunity cause susceptibility to severe COVID-19. Thus far, it is unclear to what extent specific genetic factors may explain the predisposition of individuals to developing critical COVID-19.

In the present study, we performed a GWAS involving 632 patients with critical COVID-19 to identify the potential genetic variants associated with the critical disease condition. We found that the genetic variant rs2069837 in *IL-6* was protective against critical COVID-19 (combined *p* = 4.64 × 10^−16^, OR = 0.49). IL-6 expression significantly increased in patients with critical COVID-19, and the protective GG genotype of IL-6 rs2069837 decreased the expression of IL-6 in the serum compared to its expression using the AA genotype. This finding provides a potential therapeutic target for COVID-19 based on anti-IL-6 biologics.

## Results

### Clinical features of the study subjects

In total, 632 patients with critical COVID-19 and 3021 healthy controls were included in this study. The patients with critical COVID-19 ranged in age from 20 to 75 years, and the mean age was 53.77 years. The normal healthy controls ranged in age from 40 to 72 years, with a mean age of 54.82 years (Table 1).

**Table 1:** Characteristics of the genotyped samples used for analysis

Cohort, group	Number of subjects			Age, mean (range) Years
	Total	Male	Female	
Total (Combined)	3653	1778	1875	
Discovery	2988	1396	1592	
Cases	437	225	212	58.4(6–93)
Controls	2551	1171	1380	60.7(45–89)
Replication	665	382	283	
Cases	195	112	83	61.7(22–87)
Controls	470	270	200	50.6(20–89)

The age when the cases and controls were recruited

### Significant association of genetic variants in the *IL-6* gene with COVID-19 in the Chinese population

**Fig. 1:**
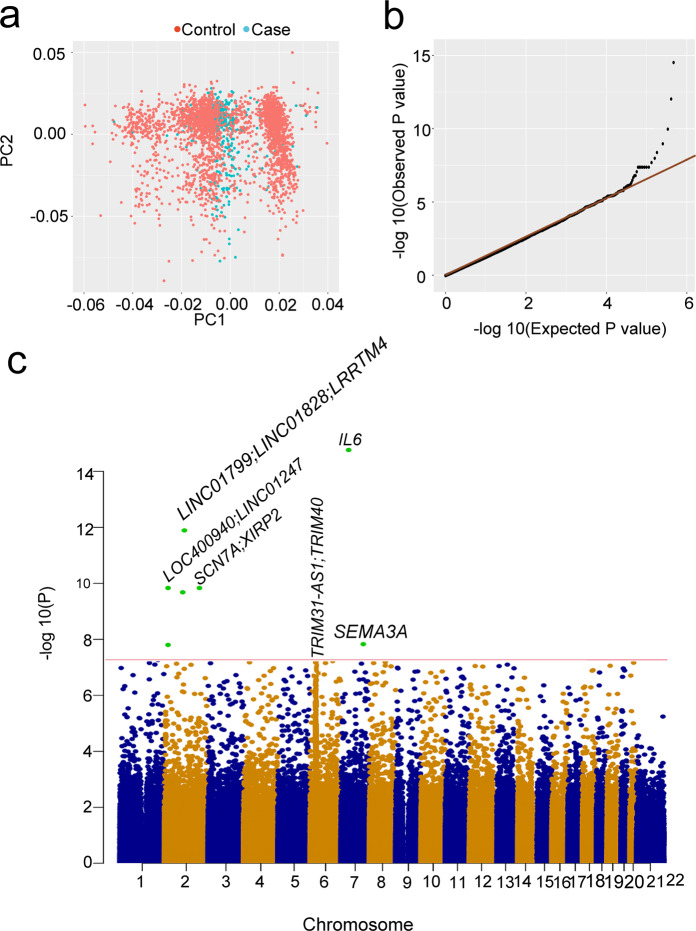
Summary of GWAS results. **a** Principal component analysis (PCA). **b** The quantile-quantile (Q-Q) plots of the GWAS results. **c** Nine loci surpassed genome-wide significance (*P* < 5 × 10^−8^) in the combined of discovery and replication results

We first tested the association of 761,993 successfully genotyped single nucleotide polymorphisms (SNPs) from 437 patients and 2551 controls. The principal component analysis (PCA, Fig. 1a) and the genomic inflation estimate (λGC) was 1.07. This indicated minimal inflation due to the population stratification of the GWAS results. We carried out a logistic test to assess the genotype-phenotype association. We compared the distribution of the observed *P* values to the distribution expected under the null hypothesis. The quantile—quantile (Q - Q) plots of the logarithms of the *P* values showed a deviation from the expected distribution, suggesting the presence of significant genetic effects (Fig. 1b).

**Fig. 2:**
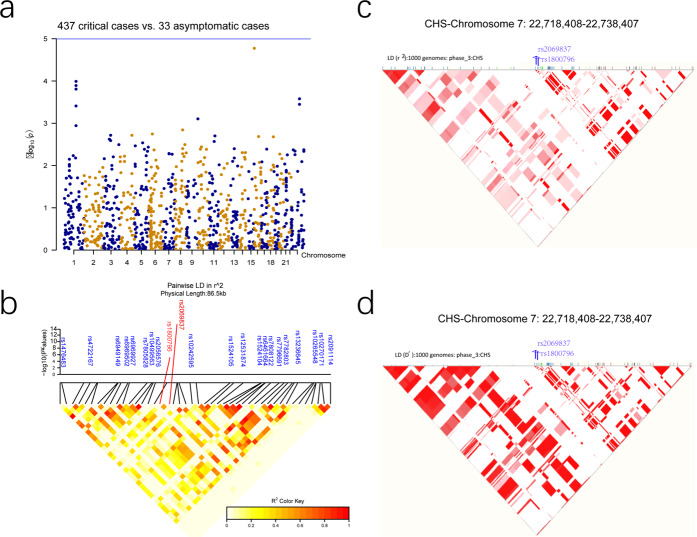
The association signal of the rs2069837 LD. **a** No loci surpassed genome-wide significance (*P* < 5 × 10^−8^) between the 437 critical and the 33 asymptomatic cases in the discovery stage of GWAS. **b** Linkage disequilibrium (LD) plot of 85.6 kb of rs2069837 association with COVID-19 in our imputed GWAS result. c-d r^2^ of LD plot of rs2069837 in 1000 Genomes Southern Han Chinese (CHS) data (**c**) and D’ of LD plot of rs2069837 in 1000 G CHS data (**d**)

A total of 10 SNPs in ten loci surpassed genome-wide significance (*p* < 5 × 10^−8^) in the discovery cohort, which comprised 437 patients with critical COVID-19 and 2,551 healthy controls (Fig. 1c and Table 2). To further validate these SNPs, we performed an LD imputation analysis and selected 19 SNPs from the same LD as that of the 10 SNPs that had reached genome-wide association significance (Table 2). Totally, nine SNPs at nine loci reached genome-wide significance (*p* < 5 × 10^−8^) using a combined discovery and replication analysis (Fig. 1c and Table 2). In the tested SNPs, no significant difference was detected between the 437 critical and the 33 asymptomatic cases. This may have been due to the small sample size in the asymptomatic cohort (Table 1 and Fig. 2a). We thus did not analyze the association between critical and asymptomatic cases further.

**Table 2:** Association results for selected SNPs in the discovery cohort and replication cohort and all cases and controls combined

SNP	CHR	BP (hg19)	A1	F_A	F_U	A2	P-Discovery	OR-Discovery	F_A	F_U	P-Replication	OR-Replication	P-meta	OR-Meta	Gene	Region
rs12995389	2	6485953	A	0.31	0.41	G	8.04 × 10^−8^	0.66	0.38	0.40	0.464	0.91	2.35 × 10^−7^	0.72	*LOC400940;LINC01247*	intergenic
rs9287675	2	6494761	A	0.24	0.32	G	5.03 × 10^−7^	0.66	0.30	0.31	0.817	0.97	3.37 × 10^−6^	0.73	*LOC400940;LINC01247*	intergenic
rs7598285	2	6505563	G	0.21	0.32	A	2.70 × 10^−12^	0.55	0.30	0.30	0.891	0.98	1.69 × 10^−10^	0.65	*LINC01247*	downstream
rs10519086	2	67262646	G	0.02	0.08	T	5.61 × 10^−11^	0.18	0.07	0.09	0.164	0.72	6.82 × 10^−11^	0.42	*LINC01799;LINC01828*	intergenic
rs72809129	2	77475663	A	0.24	0.35	C	6.97 × 10^−10^	0.59	0.43	0.47	0.268	0.87	1.42 × 10^−9^	0.67	*LRRTM4*	intronic
rs7422259	2	167377995	T	0.04	0.11	G	1.11 × 10^−9^	0.37	0.10	0.14	0.076	0.71	3.60 × 10^−10^	0.49	*SCN7A;XIRP2*	intergenic
rs4959041	6	30077967	C	0.25	0.27	T	6.90 × 10^−6^	0.92	0.23	0.25	0.279	0.86	5.88 × 10^−6^	0.91	*TRIM31-AS1*	ncRNA_intronic
rs9261445	6	30094788	T	0.23	0.26	G	4.37 × 10^−7^	0.86	0.23	0.25	0.298	0.86	5.30 × 10^−7^	0.86	*TRIM31-AS1;TRIM40*	intergenic
rs9261453	6	30099948	G	0.24	0.26	A	5.65 × 10^−6^	0.91	0.23	0.24	0.540	0.91	1.25 × 10^−5^	0.91	*TRIM31-AS1;TRIM40*	intergenic
rs9261484	6	30108683	T	0.26	0.29	C	7.14 × 10^−6^	0.89	0.24	0.26	0.492	0.91	1.33 × 10^−5^	0.89	*TRIM40*	intronic
rs9261496	6	30111014	T	0.25	0.26	C	7.88 × 10^−6^	0.91	0.22	0.25	0.410	0.89	1.11 × 10^−5^	0.91	*TRIM40*	intronic
rs9261502	6	30111610	T	0.24	0.26	A	4.51 × 10^−6^	0.91	0.22	0.24	0.568	0.92	1.11 × 10^−5^	0.91	*TRIM40*	intronic
rs9261521	6	30117773	T	0.22	0.25	C	2.61 × 10^−8^	0.81	0.24	0.25	0.860	0.97	3.17 × 10^−7^	0.85	*TRIM40;TRIM10*	intergenic
rs2069837	7	22768027	G	0.09	0.20	A	9.73 × 10^−15^	0.41	0.18	0.24	8.89 × 10^−3^	0.67	4.64 × 10^−16^	0.49	*IL6*	intronic
rs17158686	7	83794756	G	0.11	0.18	T	2.38 × 10^−7^	0.54	0.16	0.20	0.072	0.75	4.99 × 10^−8^	0.61	*SEMA3A*	intronic

*CHR* chromosome, *BP* base-pair position (hg19), *A1* Minor allele code, *A2* major allele code, *F_A* frequency of this allele in cases, *F_U* frequency of this allele in controls, *P* logistic *p* value for association test, *OR* estimated odds ratio (for A1, i.e. A2 is reference), *P-meta* *p* value for METAL combined analysis

In the initial study, we found that rs2069837 in the intronic of *IL-6* was strongly associated with COVID-19 (*p* = 9.73 × 10^−15^, Table 2). The frequency of the protective allele G was 0.09 in the patients and 0.20 in the controls. The protective allele of this SNP conferred a 0.49-fold decreased likelihood (95% confidence interval CI: 0.32–0.51) of COVID-19 (Table 2). This result was further confirmed by the replication study, in which the frequency of the protective allele G in the replication cohort (composed of 194 patients with critical COVID-19 and 471 healthy controls) differed significantly between the patients and the controls (0.1769 vs 0.2426, *p* = 8.89 × 10^−3^, OR = 0.67, 95% CI: 0.49–0.91; Table 2). In addition, a significant association between COVID-19 and rs2069837 was found when the data from the combined cohort of 632 cases and 3021 controls was analyzed (*p* = 4.64 × 10^−16^, OR = 0.49). However, we did not identify a very strong linkage association in this region (Fig. 2b) because of the week physical linkage disequilibrium between rs2069837 and other SNPs in this region in our imputed GWAS data, as well in the public 1000 genomes Southern Han Chinese (CHS) data (Fig. 2c, d).

In the combined analysis, we found that *LINC01247* rs7598285, *LINC01799* rs10519086, *LRRTM4* rs72809129, *SCN7A* rs7422259, and *SEMA3A* rs17158686 were also statistically associated with critical COVID-19 (*p* = 1.69 × 10^−10^, OR = 0.65 for rs7598285; *p* = 6.82 × 10^−11^, OR = 0.42 for rs10519086; *p* = 1.42 × 10^−9^, OR = 0.67 for rs72809129; *p* = 3.60 × 10^−10^, OR = 0.49 for rs7422259; *p* = 4.99 × 10^−8^, OR = 0.61 for rs17158686, respectively). For the reported SNPs associated with COVID-19 in previous studies^4, 7^, we found only weak associations with COVID-19 in our cohort (Supplementary Table 1).

### Lower IL-6 expression in serum in rs2069837 GG genotype compared to AA genotype and its sex-specific response

**Fig. 3:**
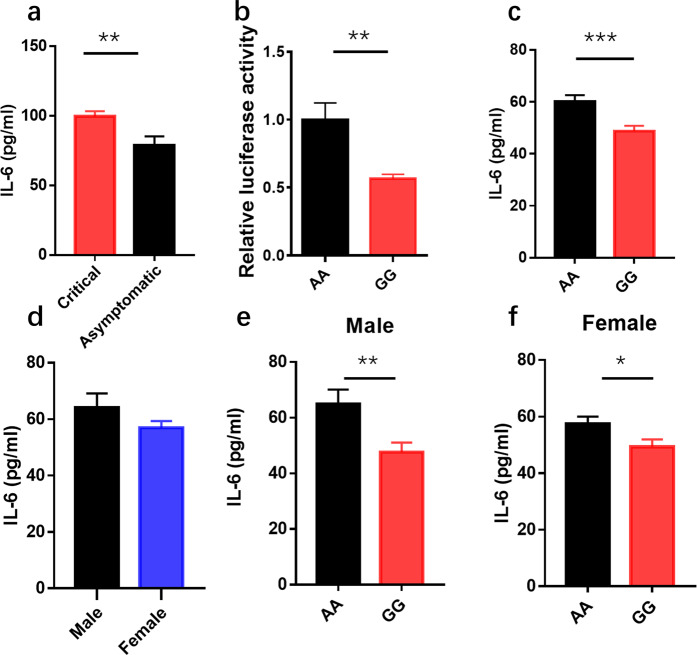
IL-6 rs2069837 region decreased its expression in the COVID-19 patients and its sex-specific. **a** Serum IL-6 concentrations between severe patients (*n* = 9) and asymptomatic patients (*n* = 34). **b** The luciferase activity of IL-6 rs2069837 region and its variation. Representative of four independent experiments with three biological replicates. **c** Serum IL-6 concentrations are shown according to IL-6 rs2069837 groups, AA type (*n* = 480), GG type (*n* = 28). **d** Baseline of IL-6 in the male (*n* = 224) and female (*n* = 284). **e** IL-6 level are shown according to IL-6 rs2069837 groups in the male, AA type (*n* = 214), GG type (*n* = 10). **f** IL-6 level are shown according to IL-6 rs2069837 groups in the female. AA type (*n* = 266), GG type (*n* = 18). Data are mean ± SEM. Significance determined by unpair T test, *P < 0.05, **P < 0.01, ***P < 0.001

In the next stage of the study, we measured the IL-6 serum of the patients with critical COVID-19 and compared it to that of asymptomatic patients. The value for IL-6 was significantly higher for the critical patients than that for the asymptomatic group (Fig. 3a), suggesting that an excessive level of IL-6 causes disease severity. Rs2069837 is located in the first intron of *IL-6*, where has promoter activity by prediction in PBMC. To analysis the effect of rs2069837 on the transcriptional activation of IL-6, we constructed a luciferase reporter system. We found that the A to G change clearly decreased the luciferase activity (Fig. 3b), which implied that the rs2069837 variant (G) could down-regulate IL-6 transcriptional activity. To further investigate the effect of rs2069837 on IL-6 secretion in the normal population, we evaluated the level of IL-6 in the serum of healthy subjects based on the IL-6 genotype (rs2069837). There were 508 individuals recruited and genotyped (480 AA types and 28 GG types of IL-6 rs2069837), and we found that the A to G variation decreased the expression of IL-6 (Fig. 3c). The baseline of IL-6 in the male and female are the same (Fig. 3d), however, the difference between AA and GG is more significant in the male than female (Fig. 3e, f, *p* = 0.007 vs *p* = 0.03).,This result revealed the genetic variant rs2069837 in IL6 has a sex-specific link to immune response. Together, our finding confirmed that the rs2069837 A to G variation may play a protective role against COVID-19 by the decreasing expression of IL-6 and inhibiting an excessive cytokine storm, especially in the male.

## Discussion

We observed a genome-wide significant association of *IL-6* rs2069837 (*p* = 9.73 × 10^−15^, OR = 0.41) with critical COVID-19 in the discovery cohort, which included 437 patients and 2,551 normal controls. When we replicated these findings in a set of 195 patients with critical COVID-19 and 470 control subjects, we detected a significant association of *IL-6* rs2069837 with COVID-19 (*p* = 8.89 × 10^−3^, OR = 0.67). When we analyzed the effect of rs2069837 on IL-6 protein expression in the serum of normal individuals based on rs2069837 genotypes, we found that the A to G change in rs2069837 decreased the expression of the IL-6 protein in the serum, particularly in the GG genotype. These results were consistent with those from a large cohort study in Wuhan that showed excessive IL-6 levels were correlated with the mortality of patients with COVID-19^8^, revealing that IL-6 may exacerbate the disease and suggesting a potential therapeutic target based on anti-IL-6 biologics.

Our GWAS also found that *LINC01247* rs7598285, *LINC01799* rs10519086, *LRRTM4* rs72809129, *SCN7A* rs7422259 and *SEMA3A* rs17158686 were significantly associated with critical COVID-19 (*p* = 1.69 × 10^−10^, OR = 0.65 for rs7598285; *p* = 6.82 × 10^−11^, OR = 0.42 for rs10519086; *p* = 1.42 × 10^−9^, OR = 0.67 for rs72809129; *p* = 3.60 × 10^−10^, OR = 0.49 for rs7422259; *p* = 4.99 × 10^−8^, OR = 0.61 for rs17158686, respectively). Of these genes, only the *SCN7A* gene was reported to be related to poliovirus infection^9^. We found that the frequency of protective allele G in *IL-6* rs2069837 was 0.09 in patients with critical COVID-19 and 0.20 in the controls in our cohort. In contrast, the frequency of allele G of this variant was 0.08 in the American and 0.09 in the European populations, according to the 1000 genomes database, which may explain why our finding for the mutation frequency in our population is different from those of several European groups for their populations. These different frequencies also provide a novel interpretation for the lower rate of criticality in the Chinese population, 13.8%, compared to 24% in the American population during the early outbreak period^10,11^. In addition, we found people carrying the GG genotype had lower IL-6 levels in our study, suggesting that those people may not easily become critical due to their low IL-6 expression when facing other cytokines storm-associated illness.

**Fig. 4:**
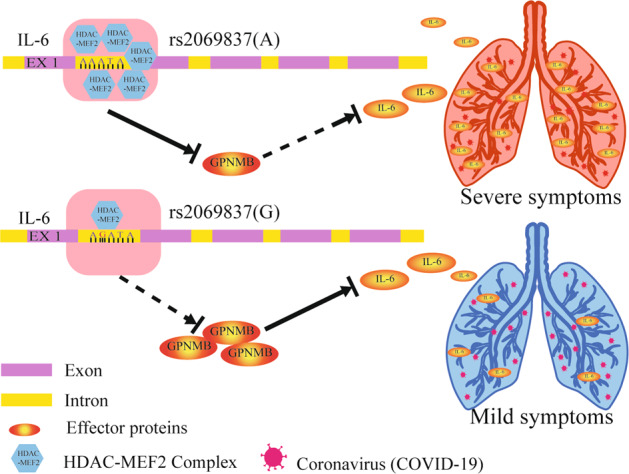
A proposed model for the regulatory mechanism of rs2069837 on the expression of IL6 involved in COVID-19. There was higher expression of MEF2 expression and lower GPNMB expression in IL6 rs2069837 AA genotype group, leading to IL6 up-regulation and severe COVID-19 development. IL6 rs2069837 GG genotype could decrease MEF2a binding and increase GPNMB expression, resulting in lower IL6 expression and prevention of severe COVID-19

In a recent study, *IL-6* rs2069837 was investigated in connection with susceptibility to hepatocellular carcinoma (HCC). There was an elevated risk from the GG genotype for HCC patients compared to the risk for healthy controls^12^. As well, studies have focused on the genetic association between Takayasu arteritis and *IL-6* rs2069837^13,14^. Their findings are consistent with ours on decreasing the expression of IL-6 in the GG genotypes. According to the prediction of potential transcription regulator of IL-6 rs2069837 in the public database, we found that MEF2 may bind to the site of IL-6 rs2069837. As the genetic variant (rs2069837 of IL6) was predicted in an enhancer region, and was found to be bond with transcription regulators -MEF2a and HDAC^14^. They demonstrated that the risk allele A of rs2069837 in IL6 represses the expression of GPNMB by recruiting MEF2-HDAC complex, which is enabled through a long-range interchromatin looping in the Takayasu arteritis. Based on our finding, allele G plays a positive role against COVID-19 by reducing IL-6, which is consistent with the function of GPNMB in that it weakens the immune response against cancer cells and infections, contributing to cancer development or chronic infections^15,16^. In our healthy cohort who carried rs2069837 AA or GG genotypes, we found that there were higher MEF2a and lower GPNMB expressions in IL6 rs2069837 AA genotype group than that in GG genotype group after pseudovirus stimulation (Supplementary Fig. 1). Taken together, we proposed a mechanism that allele G could decreased IL-6 experssion by blocked MEF2a binding and incresaed anti-inflammatory gene GPNMB (Fig. 4). We also find the impact of rs2069837 has a sex-preference link to IL-6 expression. In our COVID-19 cohort, rs2069837 displayed more significant protect role in the male than the female (*p* = 2.09^−9^, OR = 0.3557 vs *p* = 7.83^−7^, OR = 0.4451). We also confirmed males with the GG genotype had lower IL-6 baseline.

SARS-CoV-2 shows a higher mortality rate which is associated with the presence of a cytokine storm. High levels of IL-6 and Interleukin 8 (IL-8) were found in the acute stage associated with lung lesions in patients. Especially IL-6 can induce the hyper-innate inflammatory response in the respiratory tract^17,18^. SARS-CoV-2-infected patients demonstrated the up-regulation of IL-6 production and low expression of interferons^19^. Increased evidence has suggested that critically ill patients with severe respiratory failure and SARS-CoV-2 have either an immune dysregulation or a macrophage-activation syndrome, both of which are characterized by pro-inflammatory cytokines. This immune dysregulation is driven by the IL-6. There are several existing studies focused on IL-6 signaling blockading as one of the therapeutic options targeting COVID-19^20^.

In conclusion, unlike inborn error of immunity cause susceptibility to severe COVID-19, we identified IL-6 rs2069837 as a protective genetic variant in COVID-19 patients with respiratory failure and we showed that patients with the rs2069837 GG genotype had a lower level of IL-6 in their serum compared with its level for those with AA genotype.

## Materials and methods

### Subjects

We recruited 632 patients diagnosed with critical COVID-19, 33 patients with asymptomatic COVID-19, and 3,021 normal controls in this study. Among them, 158 patients critical COVID-19, all the patients with asymptomatic COVID-19 and the controls were collected from the Sichuan Provincial People’s Hospital, and 474 patients were selected from the Zhongnan Hospital of Wuhan University and the Wuhan Leishenshan Hospital. All study procedures conformed with the Declaration of Helsinki. The protocols were accepted by the Institutional Review Board and the Ethics Committee of the Sichuan Provincial People’s Hospital. We reviewed all patients’ clinical features, including signs and symptoms, the computed tomography (CT) scans of the chest, and the clinical laboratory results.

### The DNA and serum extraction

Peripheral blood was collected into 2 mL sterile EDTA tubes and stored at −80°C in a biosafety level 2 laboratory (P2 Laboratory). Genomic DNA samples were extracted from the peripheral blood using a Qiagen FlexiGene DNA kit (Qiagen, Duesseldorf, Germany). A standard serum separation protocol was followed to collect the serum from the blood samples. Specifically, the venous blood samples in the blood collection tubes without anticoagulation were allowed to clot at room temperature for 30 min (min). The serum was then obtained by centrifuge at 2000 × *g* for 10 min at 4 °C. Serum samples were flash-frozen and stored at −80 °C for further analysis. The whole separation process was completed in a P2 Laboratory.

### Genotyping and the quality control of the retained samples

The discovery cohort DNA samples were genotyped by Jinneng Biotech (Shanghai, China) using HumanOmniZhongHua-8 Bead Chips (Illumina) according to the manufacturer’s protocol, with a starting number of 900,015 SNPs. Any SNPs with a call rate of less than 90% was eliminated from further analysis. After quality filtering and cleaning, 761,993 SNPs remained for the association analysis. The SNPs that were selected for the replications were genotyped using the Sequenom Mass ARRAY system. The association analysis of the replication genotype data was conducted using PLINK 1.9, adjusted for sex.

### The association analysis

After chip genotyping, a principal component analysis (PCA) was performed separately for the disease cases and the controls to eliminate samples with outlying values from further analysis using the R statistical software package. We examined the potential genetic relatedness based on pairwise identity-by-state for all successfully genotyped samples using PLINK 1.9 software. The genomic inflation estimate (λGC) was calculated for the variants with MAF > 1% using only directly genotyped SNPs by PLINK 1.9. Single-marker association analyses were performed using PLINK 1.9 (adjusted for sex) with SNPs showing missing values <10%, MAF > 1%, and HWE *P* > 10^−6^. We used the METAL^21^ software package to perform the combined meta-analysis of the discovery and replication datasets.

### Imputation and linkage disequilibrium analysis

We performed an imputation for the 10 COVID-19-associated regions 500 kb upstream and downstream of the associated loci. The focused regional imputation was used to infer the genotypes for the SNPs that had not been directly genotyped using IMPUTE software version 2^22^ on data from the 1000 genomes database, released in June 2010. SNPs that had a quality score (Rsq) of <0.5 were discarded before the analysis. The resulting genotypes were analyzed using PLINK 1.9, as well as the same covariates for each dataset as used in the non-imputed analyses^23^. A linkage disequilibrium (LD) analysis was conducted using Haploview version 4.0 and GWAS data from the discovery cohort from the dbSNP database.

### Luciferase reporter assay and the detection of the level of IL-6

The promoter of TOPFlash (Promega, pGL4.3) was replaced with 1058 bp of IL-6 rs2069837 (in the second intron of IL-6). All plasmids were transferred into 293 T cells. After 48 h, the cells were collected and the luciferase activity was determined using the TransDetect double-luciferase reporter assay kit (TransGen, FR201-01). Blood samples were collected using coagulation-promoting tubes. The samples were centrifuged at 1500 × *g* for 15 min at room temperature, and the serum was aliquoted and stored at −70 °C. The level of IL-6 was measured using a commercial ELISA kit (mlbio, ml058097) according to the manufacturer’s protocol.

## Supplementary information

Supplementary Fig1 and Table1

## Data Availability

The datasets used and/or analyzed during the current study are available from the corresponding authors on reasonable request.
