# Supplementary material for: A genetic variant in IL-6 lowering its expression is protective for critical patients with COVID-19
Source: Signal Transduct Target Ther. 2022 Apr 2;7:112. doi: 10.1038/s41392-022-00923-1 (PMC8976167; doi:10.1038/s41392-022-00923-1)
Supplement: Supplementary file 1 — Supplementary Fig1 and Table1 [file 41392_2022_923_MOESM1_ESM.docx]

Supplementary Materials for

**A genetic variant in *IL-6* lowering its expression is protective for critical patients with COVID-19**

Bo Gong^1,2,3,4*^, Lulin Huang^2,3,4*^, Yongquan He^1,2,3,4*^, Wen Xie^5,6*^, Yi Yin^2,4^, Yi Shi^2,3,4^, Jialing Xiao^2^, Ling Zhong^2,3,4^, Yi Zhang^2,3,4^, Zhilin Jiang^2,3,4^, Fang Hao^2,3^, Yu Zhou^2,3,4^, Huan Li^2^, Li Jiang^2,3^, Xingxiang Yang^7^, Xiangrong Song^8^, Yan Kang^8^, Lin Tuo^7^, Yi Huang^2,3^, Ping Shuai^1^, Yuping Liu^1^, Fang Zheng^5#^, Zhenglin Yang^2,3,4#^

^#^Correspondence should be addressed to:

Zhenglin Yang, the Key Laboratory for Human Disease Gene Study of Sichuan Province, Sichuan Provincial People's Hospital, University of Electronic Science and Technology of China, 32 The First Ring Road West 2, Chengdu, Sichuan, 610072, China; Email: zliny@yahoo.com, Phone: 86-28-87393375, Fax: 86-28-87393596

Fang Zheng, Center for Gene Diagnosis & Department of Laboratory Medicine, Zhongnan Hospital of Wuhan University, Wuhan, China; Email: [zhengfang@whu.edu.cn](mailto:zhengfang@whu.edu.cn)


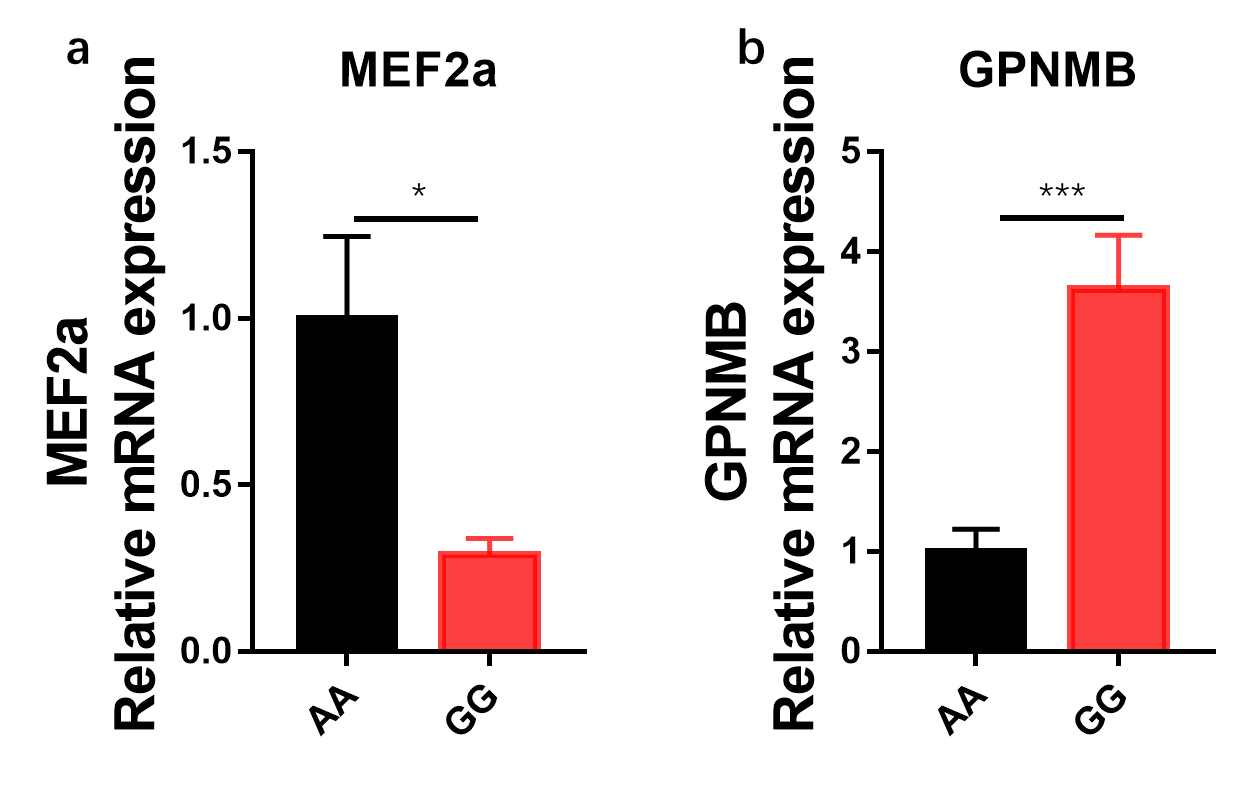
**Supplementary Fig.1** **The expression of *MEF2A* and *GPNMB* in primary PBMC cells in response of pSARS-CoV-2.** The expression of (A) MEF2a and (B) GPNMB in the PBMC 24 hours after stimulated by pSARS-CoV-2 according to IL6 rs2069837 AA or GG genotypes (n=11 for each group). Real-time PCR was used to determine the mRNA levels. The results are shown as fold changes of GG genotype relative to AA genotype. Statistical significance was determined using Welch`s correction T test. * p < 0.05, ** p < 0.01, and *** p < 0.001.

**Supplementary Table 1 Association results for SNPs in reported SNPs’LD**

| Published-SNP | SNP | LD-(r2) | LD-(D') | CHR | BP | A1 | P | OR | Gene | Region |
| --- | --- | --- | --- | --- | --- | --- | --- | --- | --- | --- |
| rs143334143 | rs67565791 | 0.57 | 0.82 | 6 | 31061013 | T | 7.15×10^-2^ | 1.337 | 10kb 3' of *U6* |  |
|  | rs2233976 | 0.78 | 0.95 | 6 | 31079994 | A | 3.22×10^-2^ | 1.467 | *C6orf15* | missense |
|  | rs113108526 | 0.82 | 1 | 6 | 31095942 | A | 8.66×10^-3^ | 1.59 | *PSORS1C1* | intronic |
|  | rs1265087 | 0.45 | 1 | 6 | 31109810 | T | 9.02×10^-4^ | 1.579 | 405bp 3' of *CCHCR1* |  |
|  | rs130072 | 1 | 1 | 6 | 31112484 | A | 7.09×10^-4^ | 1.728 | *CCHCR1* | missense |
|  | rs1265076 | 0.45 | 1 | 6 | 31113083 | T | 7.55×10^-4^ | 1.589 | *CCHCR1* | intronic |
|  | rs746647 | 0.45 | 1 | 6 | 31114182 | C | 1.37×10^-3^ | 1.556 | *CCHCR1* | intronic |
|  | rs130071 | 0.61 | 1 | 6 | 31116210 | A | 1.31×10^-3^ | 1.559 | *CCHCR1* | synonymous |
|  | rs1265114 | 0.44 | 1 | 6 | 31117188 | A | 1.40×10^-3^ | 1.555 | *CCHCR1* | intronic |
|  | rs113932411 | 1 | 1 | 6 | 31117695 | A | 7.11×10^-4^ | 1.728 | *CCHCR1* | intronic |
|  | rs1265112 | 0.45 | 1 | 6 | 31118019 | G | 2.68×10^-3^ | 1.515 | *CCHCR1* | intronic |
|  | rs2517985 | 0.45 | 1 | 6 | 31118942 | G | 4.22×10^-3^ | 1.501 | *CCHCR1* | intronic |
|  | rs17197101 | 0.92 | 1 | 6 | 31126409 | T | 3.08×10^-3^ | 1.612 | *TCF19* | 5'-UTR |
|  | rs2073724 | 1 | 1 | 6 | 31129707 | T | 6.99×10^-4^ | 1.729 | *TCF19* | missense |
|  | rs17190776 | 1 | 1 | 6 | 31130865 | A | 7.10×10^-4^ | 1.728 | *TCF19* | 3'-UTR |
|  | rs7759224 | 0.43 | 1 | 6 | 31158468 | G | 1.70×10^-5^ | 1.89 | 7.1kb 5' of *HCG27* |  |
|  | rs28362332 | 0.43 | 1 | 6 | 31162154 | A | 9.87×10^-6^ | 1.929 | 3.4kb 5' of *HCG27* |  |
|  | rs28362343 | 0.43 | 1 | 6 | 31165527 | A | 2.27×10^-6^ | 2.047 | 9bp 5' of *HCG27* |  |
|  | rs2894180 | 0.43 | 1 | 6 | 31172655 | A | 8.26×10^-6^ | 1.93 | 909bp 3' of *HCG27* |  |
|  | rs4446605 | 0.43 | 1 | 6 | 31173096 | G | 8.29×10^-6^ | 1.929 | 1.4kb 3' of *HCG27* |  |
|  | rs9263916 | 0.43 | 1 | 6 | 31176928 | C | 4.11×10^-6^ | 1.969 | 5.2kb 3' of *HCG27* |  |
|  | rs9263948 | 0.43 | 1 | 6 | 31181025 | T | 4.49×10^-6^ | 1.964 | 9.3kb 3' of *HCG27* |  |
|  | rs9263957 | 0.43 | 1 | 6 | 31184175 | A | 4.76×10^-6^ | 1.96 | 12kb 3' of *HCG27* |  |
|  | rs9263962 | 0.41 | 1 | 6 | 31185803 | A | 3.68×10^-5^ | 1.774 | 14kb 3' of *HCG27* |  |
|  | rs9263963 | 0.43 | 1 | 6 | 31186026 | T | 4.90×10^-6^ | 1.956 | 14kb 3' of *HCG27* |  |
|  | rs9263966 | 0.42 | 1 | 6 | 31186230 | T | 1.48×10^-5^ | 1.815 | 14kb 3' of *HCG27* |  |
|  | rs2394904 | 0.49 | 1 | 6 | 31216105 | A | 8.73×10^-2^ | 1.228 | 20kb 3' of *HLA-C* |  |
|  | rs4084262 | 0.53 | 1 | 6 | 31218889 | A | 4.66×10^-2^ | 1.281 | 18kb 3' of *HLA-C* |  |
|  | rs5010528 | 0.47 | 0.77 | 6 | 31241032 | G | 3.17×10^-3^ | 1.517 | 1.1kb 5' of *HLA-C* |  |
|  | rs73390981 | 0.5 | 0.77 | 6 | 31243578 | A | 4.50×10^-3^ | 1.561 | 3.7kb 5' of *HLA-C* |  |
|  | rs9461684 | 0.48 | 0.73 | 6 | 31253444 | T | 3.99×10^-4^ | 1.691 | 8.2kb 3' of XXbac-BPG248L24.13 |  |
| rs657152 | rs9411367 | 0.49 | 0.78 | 9 | 136118513 | T | 7.95×10^-3^ | 1.237 | 13kb 3' of *ABO* |  |
|  | rs9919007 | 0.56 | 0.87 | 9 | 136119527 | T | 6.12×10^-3^ | 1.25 | 12kb 3' of *ABO* |  |
|  | rs9411468 | 0.56 | 0.87 | 9 | 136119888 | A | 5.63×10^-3^ | 1.253 | 11kb 3' of *ABO* |  |
|  | rs687289 | 0.95 | 1 | 9 | 136137106 | T | 4.70×10^-3^ | 1.245 | *ABO* | intronic |
|  | rs657152 | 1 | 1 | 9 | 136139265 | T | 6.98×10^-3^ | 1.234 | *ABO* | intronic |
|  | rs507666 | 0.41 | 1 | 9 | 136149399 | A | 1.70×10^-2^ | 1.24 | *ABO* | intronic |
|  | rs630014 | 0.45 | 0.99 | 9 | 136149722 | T | 8.23×10^-2^ | 0.8693 | *ABO* | intronic |
|  | rs579459 | 0.41 | 1 | 9 | 136154168 | C | 1.64×10^-2^ | 1.24 | 3.6kb 5' of *ABO* |  |
| rs10735079 | rs10774679 | 1 | 1 | 12 | 113374748 | C | 8.52×10^-2^ | 0.8555 | *RP1-71H24.1* |  |
| rs6489867 | rs10774679 | 1 | 1 | 12 | 113374748 | C | 8.52×10^-2^ | 0.8555 | *RP1-71H24.1* |  |
| rs2109069 | rs12610495 | 0.94 | 0.99 | 19 | 4717672 | G | 1.66×10^-3^ | 1.371 | *DPP9* | intronic |

CHR: Chromosome; BP: Base-pair position (hg19); P: Logistic p-value for association test; OR: Estimated odds ratio (for A1, i.e. A2 is reference); P-meta: p-value for METAL combined analysis; LD-(r2): Linkage Disequilibrium R2; LD-(D'): Linkage Disequilibrium D’.
